# Combined Treatment with Exendin-4 and Metformin Attenuates Prostate Cancer Growth

**DOI:** 10.1371/journal.pone.0139709

**Published:** 2015-10-06

**Authors:** Yoko Tsutsumi, Takashi Nomiyama, Takako Kawanami, Yuriko Hamaguchi, Yuichi Terawaki, Tomoko Tanaka, Kunitaka Murase, Ryoko Motonaga, Makito Tanabe, Toshihiko Yanase

**Affiliations:** Department of Endocrinology and Diabetes Mellitus, School of Medicine, Fukuoka University, 7-45-1 Nanakuma, Jonan-ku, Fukuoka 814–0180, Japan; Innsbruck Medical University, AUSTRIA

## Abstract

**Introduction:**

Recently, the pleiotropic benefits of incretin-based therapy have been reported. We have previously reported that Exendin–4, a glucagon-like peptide–1 (GLP–1) receptor agonist, attenuates prostate cancer growth. Metformin is known for its anti-cancer effect. Here, we examined the anti-cancer effect of Exendin–4 and metformin using a prostate cancer model.

**Methods:**

Prostate cancer cells were treated with Exendin–4 and/or metformin. Cell proliferation was quantified by growth curves and 5-bromo–2′-deoxyuridine (BrdU) assay. TUNEL assay and AMP-activated protein kinase (AMPK) phosphorylation were examined in LNCaP cells. For *in vivo* experiments, LNCaP cells were transplanted subcutaneously into the flank region of athymic mice, which were then treated with Exendin–4 and/or metformin. TUNEL assay and immunohistochemistry were performed on tumors.

**Results:**

Exendin–4 and metformin additively decreased the growth curve, but not the migration, of prostate cancer cells. The BrdU assay revealed that both Exendin–4 and metformin significantly decreased prostate cancer cell proliferation. Furthermore, metformin, but not Exendin–4, activated AMPK and induced apoptosis in LNCaP cells. The anti-proliferative effect of metformin was abolished by inhibition or knock down of AMPK. *In vivo*, Exendin–4 and metformin significantly decreased tumor size, and further significant tumor size reduction was observed after combined treatment. Immunohistochemistry on tumors revealed that the P504S and Ki67 expression decreased by Exendin–4 and/or metformin, and that metformin increased phospho-AMPK expression and the apoptotic cell number.

**Conclusion:**

These data suggest that Exendin–4 and metformin attenuated prostate cancer growth by inhibiting proliferation, and that metformin inhibited proliferation by inducing apoptosis. Combined treatment with Exendin–4 and metformin attenuated prostate cancer growth more than separate treatments.

## Introduction

Incretin-based therapy, including dipeptidyl peptidase–4 (DPP–4) inhibitors and glucagon-like peptide–1 receptor (GLP-1R) agonists, has become a popular treatment for type 2 diabetes. Recently, much attention has been focused on incretin, because of its tissue-protective effects beyond lowering glucose levels [1]. We have demonstrated the vascular-protective effects of Exendin–4 (Ex–4), a GLP-1R agonist, as it attenuated atheroma formation in apoE^−/−^ mice via inhibition of NFkB activation in macrophages [2], and it reduced intimal thickening after vascular injury via AMP-activated protein kinase (AMPK) activation in vascular smooth muscle cells [3]. Additionally, we have recently demonstrated that the DPP–4 inhibitor, linagliptin, decreased neointima formation after vascular injury [4]. Thus, incretin-based therapy may improve the quality of life and mortality rate of patients with diabetes through its vascular-protective effects. However, cancer is another major cause of death in patients with diabetes [5], especially in Japan, where it is the leading cause of death in patients with type 2 diabetes [6]. Consequently, the Japan Diabetes Society and Japan Cancer Association have issued a warning about the increased risk of cancer in diabetic patients [7]. However, the number of studies that have examined the anti-cancer effect of incretin is limited.

Recently, we have investigated the anti-prostate cancer effect of Ex–4 both *in vivo* and *in vitro* [8]. We detected GLP-1R expression in human prostate cancer tissue and prostate cancer cell lines, and Ex–4 attenuated prostate cancer growth both *in vitro* and *in vivo* via inhibition of extracellular signal-regulated kinase-mitogen-activated protein kinase (ERK-MAPK) activation, leading to inhibition of cell proliferation [8]. As meta-analysis has suggested, the relationship between prostate cancer and diabetes or metabolic syndrome is still under discussion [9–13]. However, a recent study has suggested that pre-existing diabetes is also associated with higher mortality in patients with prostate cancer, similarly to other cancers [14]. Moreover, a follow-up study on 2,546 patients with prostate cancer has revealed that both high body-mass index and plasma C-peptide concentration increased the risk of mortality [15]. Furthermore, we have previously reported that insulin and insulin-like growth factor–1 (IGF–1) accelerate prostate cancer cell proliferation through androgen receptor (AR) activation by disrupting its direct interaction with Foxo1 [16]. These data favor the hypothesis that insulin-resistance and hyperinsulinemia in pre- or early diabetic states and metabolic syndrome are associated with poor prognosis for prostate cancer patients. However, metformin has been known as an anti-diabetic agent that also has an anti-cancer effect [17, 18]. Metformin attenuates cancer growth indirectly through reduction in serum insulin and IGF–1 concentration caused by improvement in insulin sensitivity, and directly through cell cycle arrest and inhibition of mammalian target of rapamycin (mTOR) following AMPK activation [19]. Furthermore, a detailed examination has demonstrated a direct anti-prostate cancer effect of metformin *in vivo* and *in vitro* [20].

In the present study, we examined the anti-cancer effects of Ex–4 and/or metformin treatment *in vivo* and *in vitro*, using a prostate cancer model.

## Materials and Methods

### Animals

Athymic CAnN.Cg-*Foxn1nu*/CrlCrlj non-diabetic male mice were purchased from Charles River Laboratories Japan, Inc. (Yokohama, Japan) and housed in specific pathogen-free barrier facilities at Fukuoka University. Mice were treated with either saline (n = 10) or Ex–4 (Sigma-Aldrich, Tokyo, Japan) at 300 pmol kg body weight^−1^ day^−1^ (n = 10), delivered by a mini osmotic pump (ALZEST, model 1004; DURECT, Cupertino, CA, USA), or with metformin (Wako pure chemical industries, Ltd, Osaka, Japan) at 750 mg kg^−1^ day^−1^ by mixing it with the feed (n = 10), or with combined Ex–4 and metformin (n = 10). At the age of 6 weeks, 1×10^6^ (passage 4–8) LNCaP cells were mixed with 250 μL of Matrigel (Becton Dickinson labware, Bedford, MA, USA) and transplanted subcutaneously in the flank region, and the osmotic pump was transplanted under the skin of the back of each mouse under anesthesia with 2% isoflurane inhalation. At the age of 12 weeks blood samples were collected, and mice were euthanized. One mouse treated with Ex–4 died at the age of 10 weeks, 4 days after transplantation of a new infusion pump of Ex–4, because of fighting. Tumor volume was calculated with a modified ellipsoid formula: length × width^2^ × 0.52. Paraffin-embedded formalin-fixed tumors were cut into 5-μm sections and prepared for immunofluorescent staining, as described previously [8]. Prostate serum antigen (PSA) protein concentrations in mouse serum were measured using an EIA at SRL Inc. (Tokyo, Japan). Serum insulin concentrations were measured using an EIA kit purchased from Morinaga Institute of Biological Science Inc. (Yokohama, Japan). All the procedures of animal experiments were reviewed and approved by the institutional Animal Care subcommittee at Fukuoka University Hospital.

### Cell culture And Cell proliferation assays

The human prostate cancer cell lines, LNCaP, PC3 and DU145 cells, were purchased from American Type Culture Collection (ATCC, Manassas, VA, USA). LNCaP cells and DU145 cells were maintained in RPMI 1640 medium supplemented with 10% FBS and 1% penicillin/streptomycin, PC3 cells were cultured in Ham’s F–12. All media were supplemented with 10% FBS and 1% penicillin/streptomycin. Cell proliferation assays were performed as described previously [8] with minor modifications. Briefly, cells (3×10^4^ cells/plate) were plated on 3.8-cm^2^ plates and maintained in media containing 10% FBS and 1% penicillin/streptomycin with or without 0.1–10 mM metformin, 10 nM Ex–4, a combination of 10 nM Ex–4 and 0.1 mM metformin, or 0.1 μM Compound C, an AMPK inhibitor (Sigma-Aldrich). Cell proliferation was analyzed after 0–4 days by cell counting using a hemocytometer. For all experiments, similarly passaged (passage 4–8) cells were used. Experiments were performed in triplicates using three different preparations of cells.

### siRNA Knock down of AMPK Expression and Cell Proliferation Assay

siRNA knock down and the cell proliferation assay were performed as previously described [8]. To knockdown AMPK, a possible target of metformin, we used AMPKɑ1/2 siRNA (h) (sc–45312, Santa Cruz, Santa Cruz, USA) and Control siRNA (sc–36869, Santa Cruz). For transfection, LNCaP cells were plated at a density of 1×10^5^ cells /well in 6-well plates and transfected with 10 nM AMPKɑ1/2 siRNA (h) or Control siRNA using MISSION^®^ siRNA Transfection Reagent (Sigma-Aldrich). Twenty-four hours after transfection, cells were subjected to the cell proliferation assay. Briefly, cells were detached and re-plated in 12-well tissue culture plates in complete media with or without 0.1 mM metformin. Three days after the treatment, cells were collected and counted using a hemocytometer.

### BrdU assays

To evaluate LNCaP cell proliferation, the bromodeoxyuridine (BrdU) incorporation assay was performed using Cell Proliferation ELISA kits (1647229; Roche Applied Science, Mannheim, Germany) as described previously [8]. Briefly, LNCaP cells with or without siRNA transfection were plated at 5000 cells/well in 96-well culture plates in complete media. After attaining 60–70% confluence, LNCaP cells were treated with or without 10 nM Ex–4, 0.1 mM metformin, a combination of 10 nM Ex–4 and 0.1 mM metformin, or Compound C in media with 10% FBS for 24 h. BrdU solution (10 μM) was added during the last 2 h of stimulation. Next, the cells were dried and fixed, and the cellular DNA was denatured with FixDenat solution (Roche Applied Science) for 30 min at room temperature (RT). A peroxidase-conjugated mouse anti-BrdU monoclonal antibody (Roche Applied Science) was added to the culture plates and incubated for 90 min at RT. Finally, tetramethylbenzidine substrate was added for 5 min at RT and the absorbance of the samples was measured using a microplate reader (Thermo Fisher Scientific K.K., Yokohama, Japan) at 450–620 nm. Mean data are expressed as a ratio of the control (untreated) cell proliferation.

### Apoptosis assays

For labeling nuclei of apoptotic cells, 1.5×10^5^ LNCaP cells were plated on glass coverslips in Lab-Tek Chamber Slides (177380; Nunc, Thermo Scientific, Waltham, MA, USA) and fixed in 4% paraformaldehyde for 25 min. To pretreat paraffin-embedded tissue, sections were deparaffinized, washed with xylene and ethanol and fixed in 4% paraformaldehyde for 15 min. Each section was incubated with 20 μg/mL proteinase K solution for 10 min, washed and re-fixed in 4% paraformaldehyde for 5 min. TUNEL staining was performed using the DeadEnd fluorometric TUNEL system (Promega, Madison, WI, USA) according to the manufacturer’s protocol. During the final 24 h, LNCaP cells were incubated with 0.1 or 10 mM metformin. LNCaP cells treated with 1 unit/100 μL RQ1 RNase-Free DNase (M6101; Promega) for 10 min were used as a positive control. Three independent experiments were conducted.

### Immunohistochemistry

One paraffin section was made from the center of one tumor. Paraffin sections were incubated with anti-GLP-1R antibody (NBP1-97308; Novus Biologicals, Littleton, CO, USA), anti-P504S antibody (sc–81710; Santa Cruz Biotechnology Inc.), anti-Ki67 antibody (ab66144; Abcam, Cambridge, UK), anti-AR antibody (sc–816; Santa Cruz Biotechnology Inc.) or anti-phospho-AMPKα antibody (Thr172) (#2535; Cell Signaling, Danvers, MA, USA). Sections analyzed for GLP-1Rand phospho-AMPKα (Thr172) were subsequently incubated with Alexa Fluor 488 goat anti-rabbit IgG (A–11008; Life Technologies, Carlsbad, CA, USA), and sections analyzed for P504S, AR and Ki67 were subsequently incubated with Alexa Fluor 546 goat anti-rabbit IgG (A–11010; Life Technologies). Sections were counterstained with DAPI and visualized by an LSM710-ZEN 2008 confocal microscope (Carl Zeiss Japan MicroImaging Co., Ltd., Tokyo, Japan). Four fields of one section were observed, and positive cells were counted using a hemocytometer. Data are the average of four independent count in one section.

### Western blot analysis

Western blotting was performed as described previously [8]. The following primary antibodies were used: anti-mTOR (#2983; Cell Signaling), anti-phospho-mTOR (Ser2448) (#2971; Cell Signaling), anti-phospho-AMPKɑ (Thr172) (#2535; Cell Signaling), anti-AMPKɑ (#2532; Cell Signaling) and anti-GAPDH (sc–20375; Santa Cruz). The expression of these proteins was examined in LNCaP cells that were incubated in media with 10% FBS, and subsequently stimulated with or without 10 nM Ex–4, 0.1 mM metformin, or a combination of 10 nM Ex–4 and 0.1 mM metformin for 24hr.

### Cell migration assay

Cell migration assay was performed using the CytoSelect 24-well cell migration Colorimetric Format assay (CBA-100-C; Cell Biolabs), following the manufacturer’s product manual. Each well contained a Boyden chamber with an 8-μm pore polycarbonate membrane and media containing 10% FBS with or without 10 nM Ex–4, 0.1 mM metformin, or a combination of 10 nM Ex–4 and 0.1 mM metformin. Chambers were sheeted with 1.5×10^5^ LNCaP cells or PC3 cells in serum free media and incubated at 37°C over-night. Cells within wells were washed away, and migratory cells were stained and counted using a microplate reader at OD 570 nm.

### Statistical analysis

Unpaired *t-*tests and one-way analysis of variance (ANOVA) were performed for statistical analysis as appropriate. *P* values below 0.05 were considered to be statistically significant. Results are expressed as mean ± SEM.

## Results

### Exendin–4 and Metformin Decrease Prostate Cancer Growth Additively *In Vivo*

**Fig 1:**
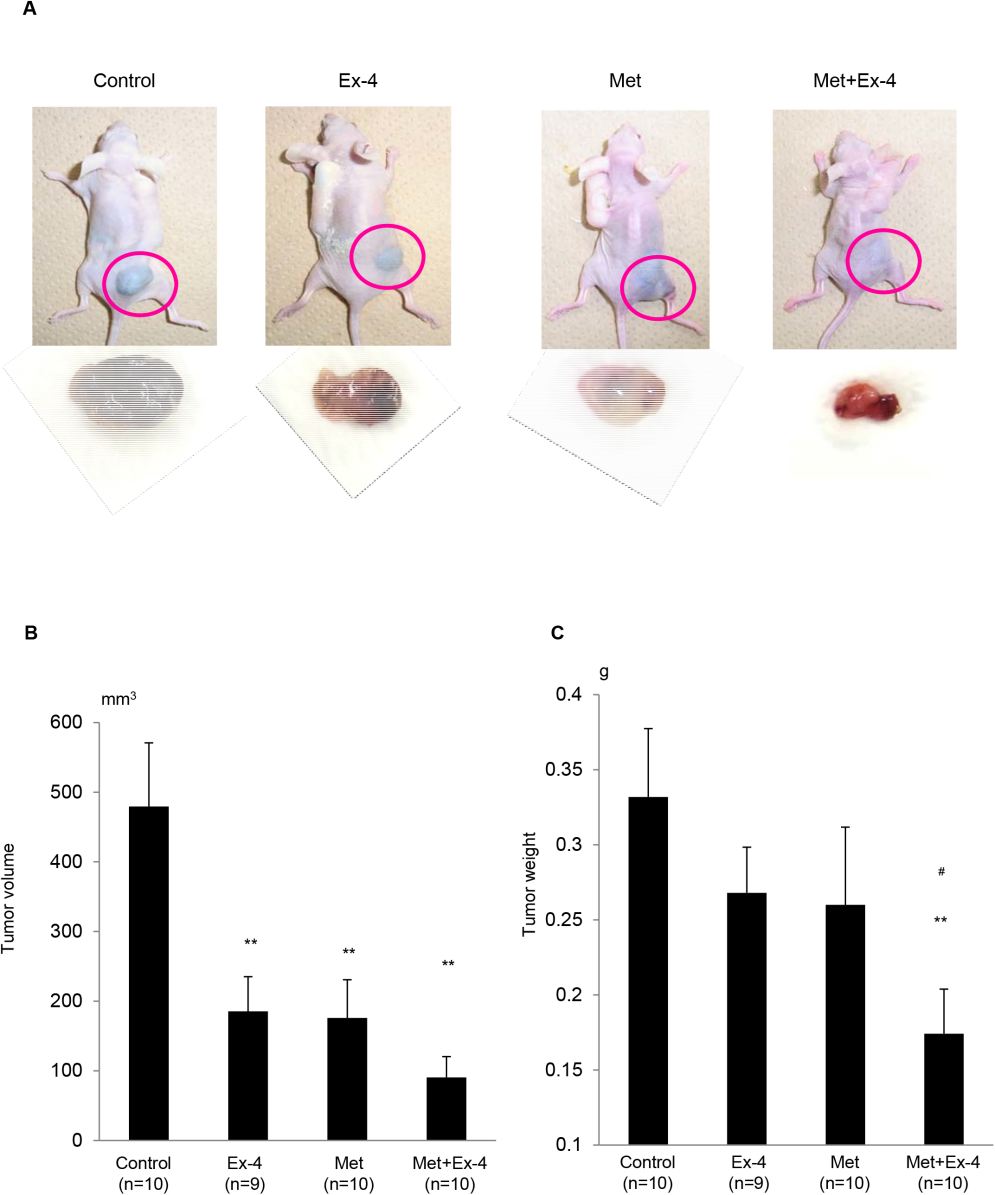
Combined treatment with Exendin–4 and metformin additively attenuates prostate cancer growth *in vivo*. (A) Athymic CAnN.Cg-*Foxn1nu*/CrlCrlj mice (aged 6 weeks) were transplanted with 1×10^6^ LNCaP cells (passage 4–8) and treated with vehicle (n = 10), Ex–4 (300 pmol kg body weight^−1^ day^−1^; n = 9), metformin (met; 750 mg kg^−1^ day^−1^; n = 10), or a combined treatment of Ex–4 and metformin (n = 10). Tumors were imaged at 12 weeks of age. (B) Tumor volume was calculated with the modified ellipsoid formula. Unpaired *t-*tests were performed to calculate statistical significance (**P < 0.01 vs. control). In the mice without a mounding tumor, tumor volume was calculated as “zero”. (C) Tumor weight was measured on scales. Unpaired *t*-tests were performed to calculate statistical significance (**P < 0.01 vs. control; ^#^ *P* < 0.05 vs. Ex–4). In the mice without a mounding tumor, tumor weight was calculated as “zero”.

We treated athymic mice, which were transplanted with LNCaP cells, with subcutaneously administered Ex–4, orally fed metformin or the combined treatment. After 6 weeks of treatment, tumor mounding was absent in one mouse of the Ex-4-treated group, two mice of the metformin-treated group and three mice of the combined treatment group. Calculation of tumor size using the modified ellipsoid formula revealed that the tumor volume significantly decreased after treatment with both Ex–4 and metformin compared with the control, but further reduction in tumor size was observed in the combined treatment mice (Fig 1A and 1B). Measurement of the weight of the formed tumors demonstrated that the combined treatment with Ex–4 and metformin significantly reduced tumor weight compared with that of the control and of the separate treatment with Ex–4 or metformin (Fig 1C). These data suggest that Ex–4 and metformin attenuated prostate cancer growth *in vivo*, and the combined treatment with both of them further decreased tumor growth additively.

In these mice, the final body weight and plasma glucose level were significantly lower in the metformin-treated group compared with the control and Ex-4-treated groups (Table 1). The plasma PSA level slightly decreased after Ex–4 or metformin treatment alone compared with the control, and a further and significant reduction was observed after the combined treatment compared with the control (Table 1). Ex–4 significantly increased the serum insulin level; however, the combined Ex–4 and metformin treatment decreased it to the control level (Table 1).

**Table 1:** Characteristics of treated athymic mice following transplantation of LNCaP cells.

	Control(n = 10)	Ex–4(n = 9)	Metformin(n = 10)	Ex–4 and metformin(n = 10)
Body weight(g)	23.4±0.8	24.0±0.6	19.3±0.5**,^##^	19.4±0.6**,^##^
Plasma glucose(mg/dL)	153.6±6.5	147.4±12.7	85.3±8.4**,^##^	80.4±10.2**,^##^
Serum insulin(ng/mL)	0.17±0.02	0.43±0.1*	0.16±0.04^#^	0.16±0.1^#^
Plasma PSA(ng/mL)	7.34±1.8	3.06±0.7	4.68±1.6	1.12±0.6*

Data are mean ± SEM. One-way ANOVA was performed to calculate statistical significance (*P < 0.05, **P < 0.01 vs. control, ^#^ *P* < 0.05, ^##^ *P* < 0.01 vs. Ex–4).

### Exendin–4 and Metformin Decrease Cell Proliferation and Increase GLP-1R Expression

**Fig 2:**
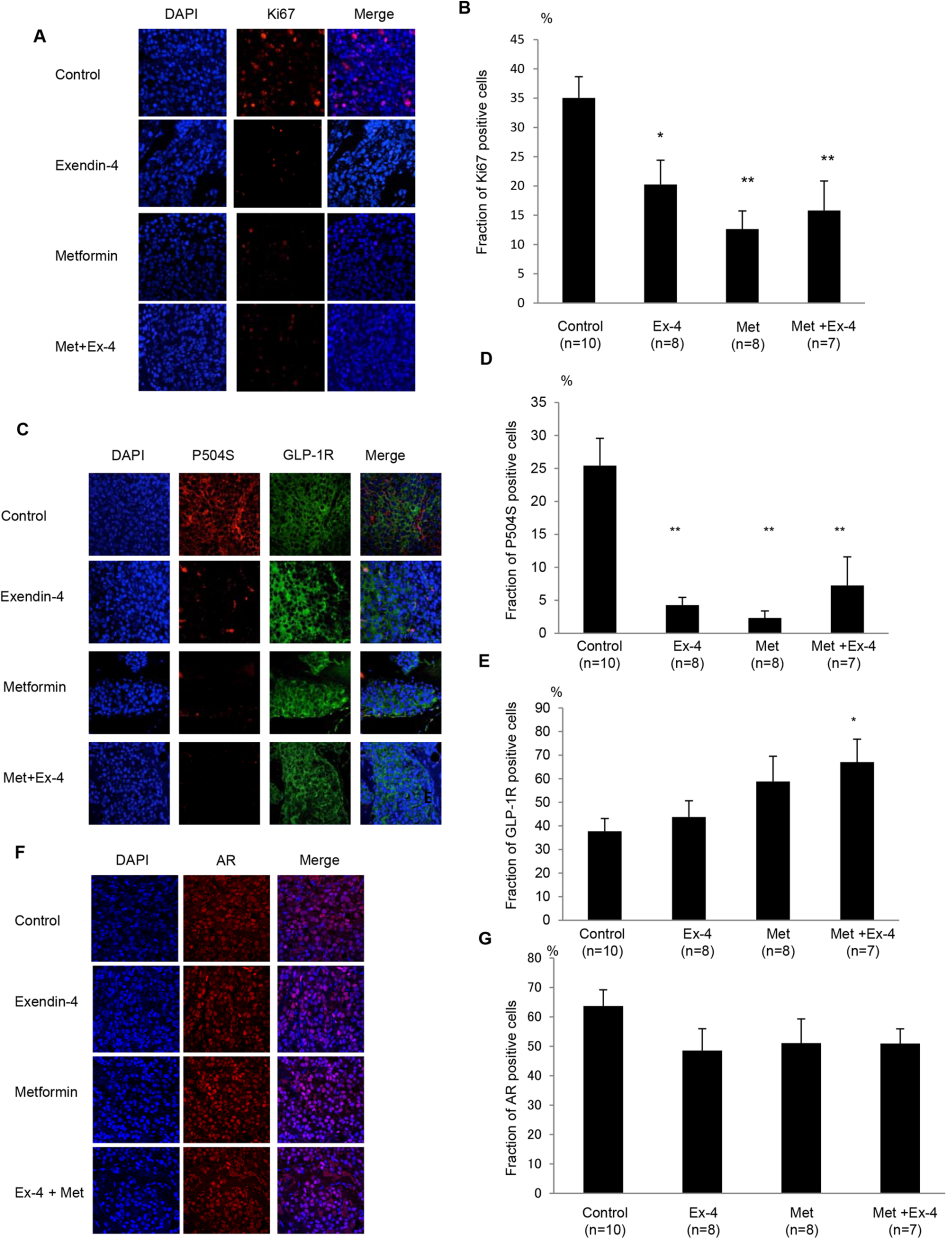
Exendin–4 and metformin decrease the number of Ki67 and P504S-positive cells, and increase GLP-1R expression. Paraffin-embedded tumor sections (5 μm) were subjected to immunohistochemistry for (A) Ki67, (C) P504S and GLP-1R, and (F) AR, and counterstained with DAPI. Magnification, ×400. (B) Ki67, (D) P504S, (E) GLP-1R, and (G) AR-positive cells were quantified by analyzing the fraction of stained cells in the tumor relative to the total number of nuclei. Values are expressed as a percentage of positive cells. Unpaired *t*-tests were performed to calculate statistical significance (*P < 0.05, **P < 0.01 vs. control).

Immunohistochemical analysis of paraffin-embedded sections of subcutaneous prostate cancer tumors demonstrated that Ki67 expression, which was clearly localized within the nucleus, was significantly suppressed by Ex–4, metformin and the combined treatment (Fig 2A and 2B). However, an additional decrease in Ki67-positive cells was not observed in the combined treatment group compared with the separate treatments. The expression of P504S, a prostate cancer marker, dramatically decreased by Ex–4, metformin and the combined treatment (Fig 2C). Quantification of P504S expression based on the mean number of P504S-positive cells divided by the total number of nuclei confirmed that there was a significant reduction in cancerous cells after Ex–4, metformin and the combined treatment (Fig 2D). Interestingly, the number of prostate cancer cells expressing GLP-1R increased after Ex–4 and/or metformin treatment (Fig 2C). Quantification of the proportion GLP-1R-positive cells revealed that the combined treatment significantly increased the number of GLP-1R-positive cells compared with the control (Fig 2E). Furthermore, we detected AR-positive cells in the prostate cancer tumor (Fig 2F). No change was observed in AR expression after Ex–4 and metformin treatment in the prostate cancer tumor (Fig 2G).

### Exendin–4 and Metformin Attenuate Prostate Cancer Cell Proliferation, but not Cell Migration

**Fig 3:**
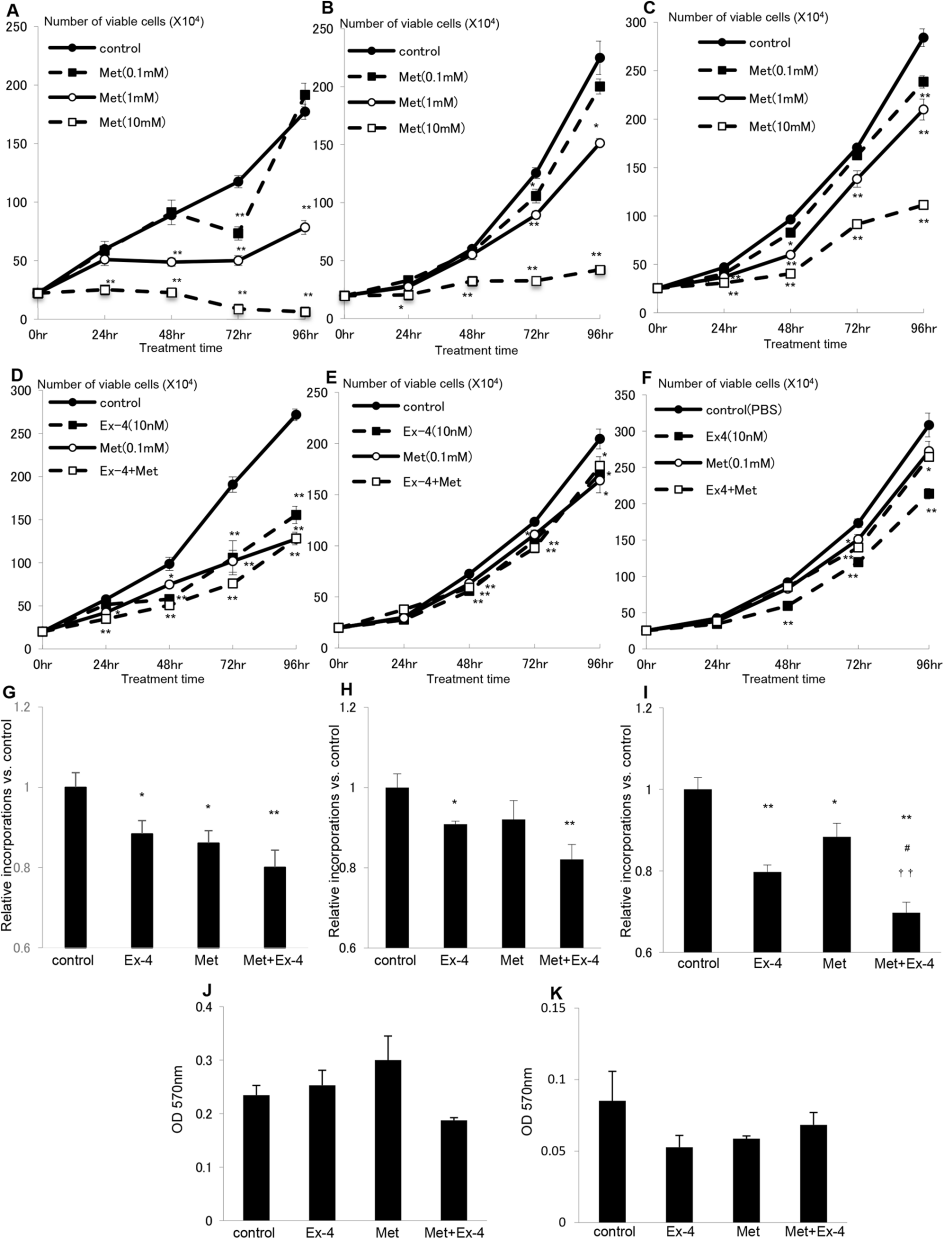
Exendin–4 and metformin inhibit prostate cancer cell proliferation additively, but not migration. (A) LNCaP cells, (B) PC 3 cells and (C) DU145 cells were maintained in media supplemented with 10% FBS with or without metformin (0.1–10mM). After 0, 24, 48, 72 and 96 h, the cells were harvested, and cell proliferation was analyzed by cell counting using a hemocytometer. Unpaired *t*-tests were performed to calculate statistical significance (*P < 0.05, **P < 0.01 vs. control). (D) LNCaP cells, (E) PC3 cells and (F) DU145 cells were maintained as described in A-C, but with the indicated treatments. Unpaired *t*-tests were performed to calculate statistical significance (*P < 0.05, **P < 0.01 vs. control). (G) LNCaP cells, (H) PC3 cells and (I) DU145 cells were plated at a density of 5000 cells/well in 96-well plates in media supplemented with 10% FBS and incubated with saline (control), Ex–4 (10nM), metformin (0.1mM), or both Ex–4 (10nM) and metformin (0.1mM) for 24 h. BrdU solution was added during the last 2 h, and cells were harvested for measurement of DNA synthesis using a microplate reader at 450–620 nm. Mean data are expressed as a ratio of the control cell proliferation. Unpaired *t*-tests were performed to calculate statistical significance (*P < 0.05, **P < 0.01 vs. control, ^#^ *P* < 0.05 vs. Ex–4, ^††^ *P* < 0.01 vs. metformin). (J) LNCaP cells and (K) PC3 cells were seeded as 1.5×10^5^ in each chambers for the migration assay. After chemo attraction with 10% FBS with or with saline (control), Ex–4 (10 nM), metformin (0.1 mM) or both Ex–4 (10 nM) and metformin (0.1 mM), cells were stained and examined at OD 570nm. Unpaired *t*-tests were performed to calculate statistical significance.

We next examined the effect of Ex–4 and metformin on prostate cancer cells *in vitro*. In our previous study [8], we observed that Ex–4 significantly reduced the cell number of the human androgen-dependent cells, LNCaP cells, and of the human androgen-independent cells, PC3 cells and DU145 cells, in growth curves in a dose-dependent manner. Similar to the Ex–4 treatment, metformin reduced the cell number of LNCaP cells (Fig 3A), PC3 cells (Fig 3B) and DU 145 cells (Fig 3C) in a dose-dependent manner. Furthermore, the combined treatment of 0.1 mM metformin and 10 nM Ex–4 additively attenuated the growth curve progression of LNCaP cells (Fig 3D) and PC3 cells (Fig 3E), but not of DU145 cells (Fig 3F). We next examined the mechanism by which Ex–4 and metformin inhibit prostate cancer cell growth. First, we performed a BrdU incorporation assay to assess DNA synthesis. Ex–4 and metformin treatment alone for 24 h significantly decreased DNA synthesis in LNCaP cells (Fig 3G). Although treatment with metformin alone did not cause a statistically significant reduction in BrdU incorporation in PC3 cells (Fig 3H), Ex–4 and metformin decreased BrdU incorporation in both PC3 cells and DU145 cells (Fig 3I), similarly to LNCaP cells. The combined treatment of metformin and Ex–4 further decreased BrdU incorporation, suggesting that metformin and Ex–4 additively decreased DNA synthesis in prostate cancer cells (Fig 3G–3I). Furthermore, we performed a migration assay on LNCaP cells and PC3 cells. However, Ex–4, metformin and the combined treatment did not attenuate cell migration in LNCaP cells (Fig 3J). In PC3 cells, a small reduction by treatments was observed, but it was not statistically significant (Fig 3K). These data suggest that Ex–4 and metformin attenuates prostate cancer cell proliferation, but not cell migration.

### Metformin induces apoptosis and attenuates cell proliferation in prostate cancer cells via AMPK activation

**Fig 4:**
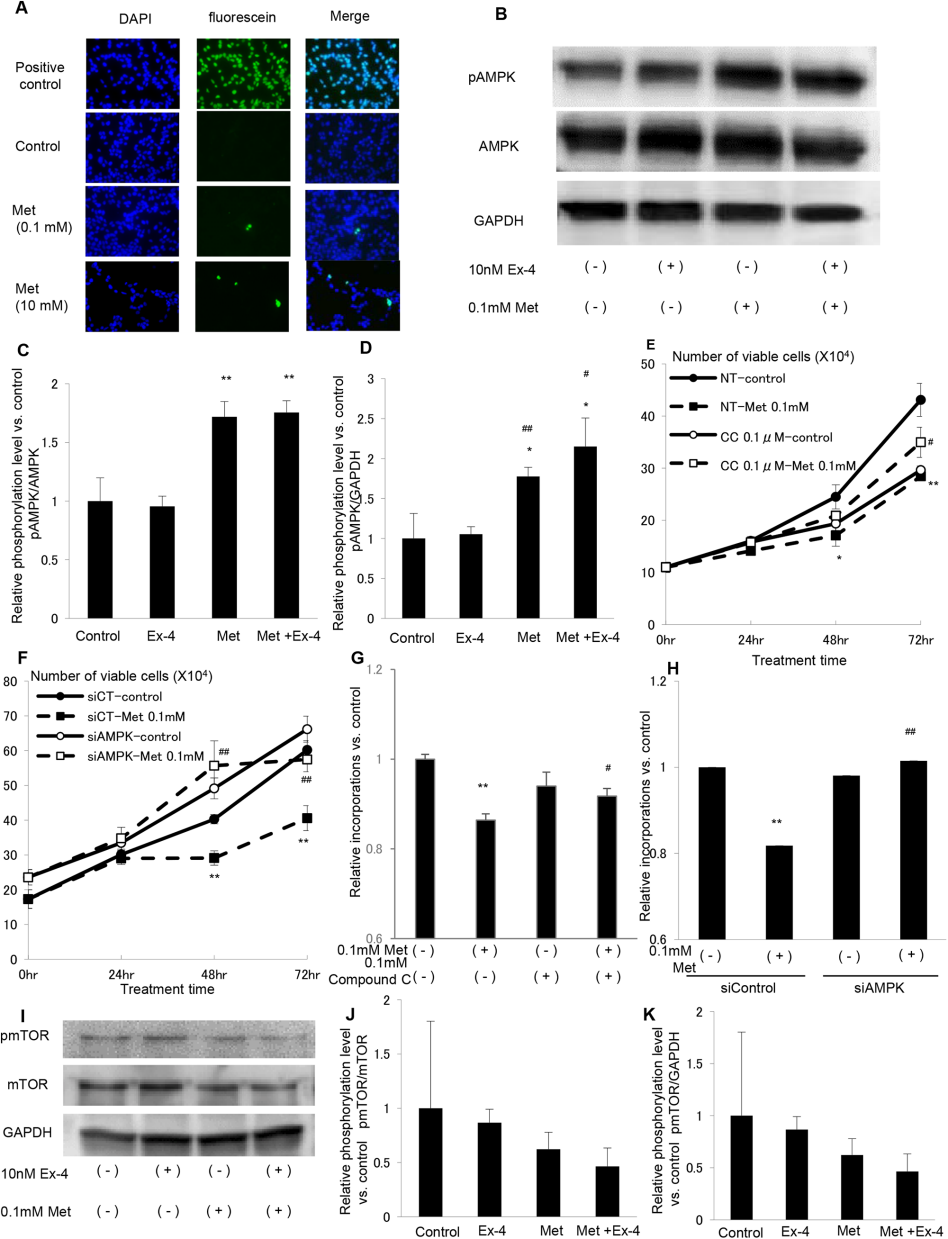
Metformin induces apoptosis and attenuates cell proliferation in prostate cancer cells via AMPK activation. (A) LNCaP cells were plated on glass coverslips in Lab-Tek 2 well Chamber Slides. After incubation with 0.1 or 10 mM metformin for 24 h, or 1 unit/100 μL RQ1 DNase (positive control) for 10 min, apoptotic cells were detected with TUNEL staining. Images shown are representative of three independent experiments. (B) LNCaP cells maintained in media with 10% FBS were stimulated with saline (control), Ex–4 (10 nM), metformin (0.1 mM) or both Ex–4 (10 nM) and metformin (0.1 mM) for 24 h. Cell lysates were harvested and subjected to western blotting to assess phosphorylated AMPK and AMPK expression. Phosphorylated AMPK/AMPK protein levels (C) and phosphorylated AMPK/GAPDH protein levels (D) were quantified by densitometry. Data were calculated from three independent experiments and are shown as a ratio of the control (*P < 0.05, **P < 0.01 vs. control, ^#^ *P* < 0.05, ^##^ *P* < 0.05 vs. Ex–4). (E) LNCaP cells were maintained in media supplemented with 10% FBS with Compound C (0.1μM; CC) or control vehicle (NT), and with or without Met (0.1mM). After 0, 24, 48 and 72h, the cells were harvested, and cell proliferation was analyzed by cell counting using a hemocytometer. Unpaired *t*-tests were performed to calculate statistical significance (*P < 0.05, **P < 0.01 vs. NT-control, ^#^ *P* < 0.05 vs. NT-Met 0.1 mM). (F) LNCaP cells were maintained in media supplemented with 10% FBS after transfection of 10 nM control siRNA (siCT) or siRNA for AMPKα1/2 (siAMPK) with or without Met (0.1 mM). After 0, 24, 48 and 72h, the cells were harvested, and cell proliferation was analyzed by cell counting using a hemocytometer. Unpaired *t*-tests were performed to calculate statistical significance (**P < 0.01 vs. siCT-control, ^##^ *P* < 0.01 vs. siCT-Met 0.1 mM). (G) LNCaP cells were plated at a density of 5000 cells/well in 96-well plates in media supplemented with 10% FBS and incubated with Compound C (0.1μM) (CC) or control vehicle (NT), and with or without Met (0.1mM) for 24 h. BrdU solution was added during the last 2 h, and cells were harvested for measurement of DNA synthesis using a microplate reader at 450–620 nm. Mean data are expressed as a ratio of the control cell proliferation. Unpaired *t*-tests were performed to calculate statistical significance (**P < 0.01 vs. Met (-) and Compound C (-), ^#^ *P* < 0.05 vs. Met (+) and Compound C (-)). (H) LNCaP cells were plated at a density of 5000 cells/well in 96-well plates in media supplemented with 10% FBS after transfection of 10nM control siRNA (siCT) or siRNA for AMPKɑ1/2 (siAMPK) with or without Met (0.1mM) for 24h. BrdU solution was added during the last 2 h, and cells were harvested for measurement of DNA synthesis using a microplate reader at 450–620 nm. Mean data are expressed as a ratio of the control cell proliferation. Unpaired *t*-tests were performed to calculate statistical significance (**P < 0.01 vs. Met (-) and siControl, ^##^ *P* < 0.01 vs. Met (+) and siControl).

We next examined apoptosis using the TUNEL assay. Although apoptotic cells were not observed in Ex-4-treated LNCaP cells in our previous study [8], a small but significant number of apoptotic cells was detected in 0.1 or 10 mM metformin-treated LNCaP cells (Fig 4A). Because AMPK activation is one of the most important molecular mechanisms by which metformin acts as a metabolic and anti-proliferative agent [21], we examined AMPK phosphorylation in LNCaP cells treated with metformin. As shown in Fig 4B, AMPK was phosphorylated after metformin treatment. Quantification of the detected band densitometry revealed a significant induction of AMPK phosphorylation in metformin-treated LNCaP cells when normalized against total AMPK (Fig 4C) and the house-keeping gene, GAPDH (Fig 4D). To clarify whether AMPK is the main target of metformin for attenuation of LNCaP cell proliferation, we used the AMPK inhibitor, Compound C, or knocked down AMPK by siRNA. As shown in Fig 4E and 4F, both Compound C and siAMPK clearly cancelled the anti-proliferative effect of metformin on LNCaP cells. In addition, treatments with Compound C or siAMPK significantly increased the cell number of metformin treated LNCaP cells. Furthermore, the reduction in BrdU incorporation induced by metformin was clearly abolished by both Compound C and siAMPK (Fig 4G and 4H), and these treatments increased BrdU incorporation in metformin-treated LNCaP cells in consistently with the growth curve analysis. Because the main target of the anti-proliferative effect of AMPK is inactivation of mTOR, we next examined mTOR activation using western blotting. As shown in Fig 4I, mTOR phosphorylation was supressed by metformin treatment. However, densitometry analysis showed that result was not statistically significant. These data suggest that metformin induces apoptosis and attenuates LNCaP cell proliferation via AMPK activation.

### Metformin Induces AMPK Activation and Apoptosis in Prostate Cancer *In Vivo*

**Fig 5:**
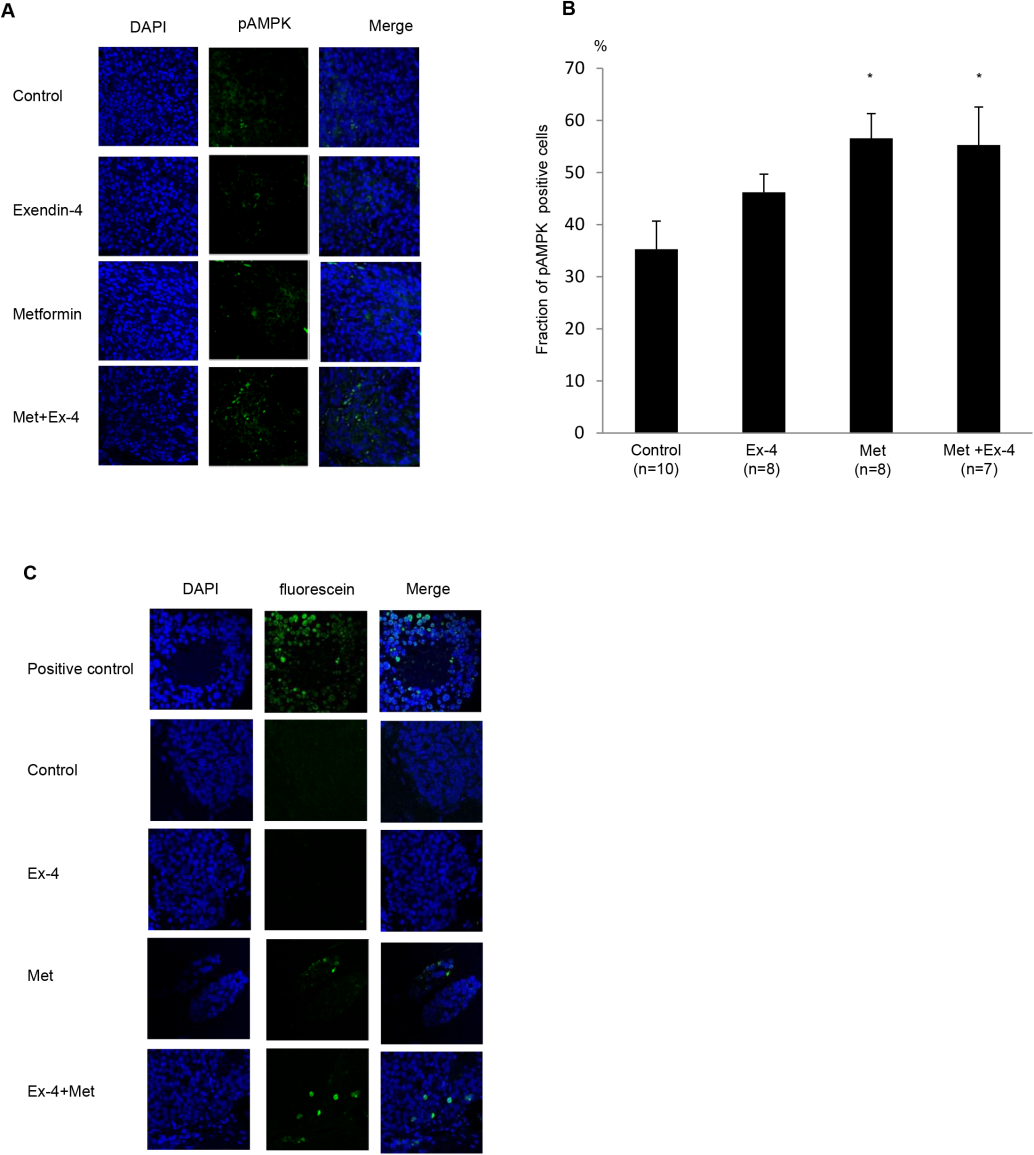
Metformin induces AMPK activation and apoptosis in prostate cancer *in vivo*. (A) Sections (5 μm) were subjected to immunohistochemistry for phospho-AMPK and counterstained with DAPI. Magnification, ×400. (B) Phospho-AMPK-positive cells were quantified by analyzing the fraction of stained cells in the tumor relative to the total number of nuclei. Values are expressed as a percentage of positive cells. Unpaired *t-*tests were performed to calculate statistical significance (*P < 0.05 vs. control). (C) Sections (5 μm) were subjected to apoptosis assay. Sections from tumors of untreated control mice were incubated with 1 unit/100 μL RQ1 DNase (positive control) for 10 min. Apoptotic cells were detected by TUNEL staining. Images shown are representative of three independent experiments.

Finally, to confirm the mechanism elucidated from the *in vitro* experiments, we examined AMPK phosphorylation and apoptosis in prostate cancer tumors formed in athymic mice. As shown in Fig 5A and 5B, AMPK phosphorylation also increased after metformin treatment *in vivo*, which is consistent with the *in vitro* experiment. Quantification of phospho-AMPK-positive cells divided by the total number of nuclei confirmed that there was significant AMPK activation after metformin treatment compared with the control. Furthermore, the TUNEL assay revealed that metformin treatment, but not Ex–4, induced apoptosis in prostate cancer *in vivo* as well (Fig 5C). Taken together, these data suggest that metformin decreased prostate cancer growth not only by inhibiting cell proliferation, but also by inducing apoptosis through AMPK activation.

## Discussion

In the present study, we demonstrated that the GLP-1R agonists, Ex–4 and metformin, significantly and additively decreased prostate cancer growth. The mechanisms underlying this anti-cancer effect may be attenuation of cell proliferation by both Ex–4 and metformin, in addition to induction of apoptosis by metformin through AMPK activation. However, we did not observed an anti-migration effect of Ex–4 and metformin in the present study.

Incretin-based therapy is a recently established anti-diabetic therapy [22]. It has become very popular very quickly throughout the world, including Japan [23], because it has many benefits, such as pancreatic beta cell preservation, reduced appetite and deceleration of gastric emptying, and improvement of insulin sensitivity [24]. Additionally, incretin-based therapy has been developed as an anti-diabetic agent aimed to decrease the blood glucose level in patients with type 2 diabetes, while its pleiotropic tissue-protective effects have also been demonstrated [1]. In our previous study, we have demonstrated the anti-prostate cancer effect of Ex–4 as a newly identified benefit of incretin-based therapy [8].

Following our previous report, here we demonstrated by *in vivo* and *in vitro* experiments that a stronger anti-prostate cancer effect could be achieved by combining Ex–4 with metformin treatment compared with the treatments alone. Metformin is well known as an anti-cancer agent [18, 19], including prostate cancer [25]. Although the main molecular target of metformin is AMPK [26], several AMPK-dependent and independent mechanisms by which metformin inhibits prostate cancer growth have been reported, such as suppression of cyclin D1 expression [20], mTOR inhibition [27], inhibition of androgen-induced IGF–1 receptor up-regulation [28], and inhibition of lipogenesis [29]. Additionally, an interesting study suggesting a relationship between ERK-MAPK and metformin has been reported. Inhibition of p42 MAPK, an isoform of ERK-MAPK, using siRNA potentiated the anti-prostate cancer effect of metformin [30]. This report is consistent with our present study. We have previously observed ERK-MAPK inactivation by Ex–4 [8], and further inhibition of prostate cancer growth was observed after the combined treatment with Ex–4 and metformin. In the present study, we observed that metformin decreased mTOR phosphorylation (Fig 4I). However, this was not statistically significant (Fig 4J and 4K) and not as drastic as the metformin-induced anti-proliferative effect on the growth curve (Fig 4A–4F). Hence, another mechanism may exist. Furthermore, LKB1, an AMPK activator, was not phosphorylated in metformin-treated LNCaP cells (data not shown). Thus, another mechanism, by which metformin activates AMPK, may be involved in prostate cancer cells.

In the *in vivo* experiments, further reduction in tumor size and volume was observed after the combined treatment compared with Ex–4 or metformin treatment alone (Fig 1). However, the data obtained from Ki67 staining suggest that further reduction in cell proliferation was not achieved by the combined treatment compared with the treatments alone (Fig 2A and 2B). In contrast, apoptosis assays revealed that metformin, but not Ex–4, induced apoptosis in prostate cancer both *in vivo* and *in vitro* (Figs 4A and 5A). These data suggest that the increased reduction in prostate cancer size and volume by adding metformin to Ex–4 treatment was caused by apoptosis induction. As we have previously reported, Ex–4 does not induce apoptosis in prostate cancer cells [8]. The present data suggest that metformin potentiates the anti-prostate cancer effect of Ex–4 by inducing apoptosis. Additionally, Ex–4 increased the serum insulin level (Table 1), probably because incretin is an insulin secretagogue [31]. However, combining Ex–4 with metformin significantly decreased the serum insulin level to a level similar to the control level (Table 1). The reduction in the insulin level may be one of the mechanisms by which metformin additively decreased prostate cancer tumor size *in vivo*, because insulin is one of the growth factors of prostate cancer cells, as we have previously demonstrated [16]. Furthermore, the combined treatment with Ex–4 and metformin significantly increased GLP-1R expression in prostate cancer *in vivo* (Fig 2E). Previously it has been reported that Ex–4 increased GLP-1R expression in glomeruli of diabetic and non-diabetic mice [32]. Additionally, it has also been reported that metformin increased GLP-1R expression in pancreatic beta cells in a peroxisome proliferator-activated receptor-α-dependent manner [33]. Actually, both Ex–4 and metformin slightly increased GLP-1R expression, although it was not statistically significant. Up-regulation of GLP-1R induced by the combined treatment with Ex–4 and metformin may be one of the mechanisms by which Ex–4 and metformin additively attenuate prostate cancer growth.

In the present study, there are several limitations in our experimental design. First, we transplanted prostate cancer cells into non-diabetic male mice, because we wanted to examine the anti-tumor effect of Ex–4 and metformin independently of the glucose lowering effect. However, these anti-diabetic agents are provided to patients with diabetes that have been in a hyperglycemic state. Whether the anti-cancer effect of Ex–4 and metformin demonstrated in the present study is reproducible in the diabetic state was not confirmed. Because other data have suggested that hyperglycemia reduced GLP-1R expression in pancreatic beta cells [34], GLP-1R expression in tumors may also be decreased in the hyperglycemic state. Further elucidation with diabetic models is required. Second, we only used LNCaP cells in the *in vivo* experiments, following our previous report [8]. In the *in vitro* experiments, we observed the anti-proliferative effects of Ex–4 and metformin on other prostate cancer cell lines, PC3 cells and DU145 cells. In fact, LNCaP cells are not so adhesive cells compared with other prostate cancer cell lines possibly because of galectin expression [35]. In addition, we treated the mice with drugs simultaneously with LNCaP cell transplantation, without confirmation of tumor mounding. Thus, the absence of tumor mounding in one Ex-4-treated mouse, two metformin-treated mice and three combined-treated mice may be caused not only by the anti-cancer effect of these drugs, but also because of technical failure or the character of LNCaP cells.

In the recently announced updated positional statement of the American Diabetes Association and the European Association for the Study of Diabetes (EASD) for management of hyperglycemia in type 2 diabetes, metformin was recommended as first-line therapy, and combined therapy of metformin and incretin-based therapy, such as GLP-1R agonists and DPP–4 inhibitors, was recommended as second-line therapy [36]. The combined therapy of metformin and incretin-based therapy has several benefits, such as a lower risk of weight gain and hypoglycemia [37]. The present study demonstrated a new benefit of this combination therapy, an anti-prostate cancer effect.

## Abbreviations

AMPK: AMP-activated protein kinase
AR: androgen receptor
DPP–4: dipeptidyl peptidase–4
Ex–4: Exendin–4
ERK-MAPK: extracellular signal-regulated kinase-mitogen-activated protein kinase
GAPDH: glyceraldehyde 3-phosphate dehydrogenase
GLP–1: glucagon-like peptide–1
GLP-1R: GLP–1 receptor
IGF–1: insulin-like growth factor-1
mTOR: mammalian target of rapamycin
NFkB: nuclear factor kappa-light-chain-enhancer of activated B cells
PSA: prostate serum antigen
